# Zeitotox: Toxicology and the Rhythms of Life

**DOI:** 10.1021/acs.est.2c02961

**Published:** 2022-07-27

**Authors:** Linhong Xiao, Philipp Antczak, Joëlle Rüegg, Lars Behrendt

**Affiliations:** † Science for Life Laboratory, Department of Organismal Biology, Uppsala University, Norbyv. 18A, 75236 Uppsala, Sweden; ‡ Center for Molecular Medicine Cologne, Lab. of Computational Biology of Ageing, University of Cologne, Robert-Koch-Str. 21, 50931 Cologne, Germany

**Keywords:** environmental toxicology, physiological rhythms, phytoplankton, systems toxicology, human-derived cells

Physiological rhythms set the cadence of life. In humans, physiological rhythms mediate sleeping, waking, menstrual cycles, and metabolism, whereas migration, photosynthesis, and hibernation are mediated by periodic oscillations in “natural” organisms. Rhythms are not isolated but interact with each other as well as their extrinsic environment, chiefly to match individual and population physiologies to diel or lunar cycles or changing seasons. The environment and individual organisms’ physiologies are thus deeply connected, albeit this correlation is often overlooked when studying organisms in the context of medicine and environmental toxicology. In this opinion paper, we advocate for the formal inclusion of externally mediated physiological rhythms into the study of environmental toxicology. To highlight this need, we feature two systems where physiological rhythms play a fundamental role, yet little is known about how the timing of chemical exposures might affect their physiological outcomes.

## Rhythms That Feed the Planet: Light, Photosynthesis, and Plankton

**Figure 1:**
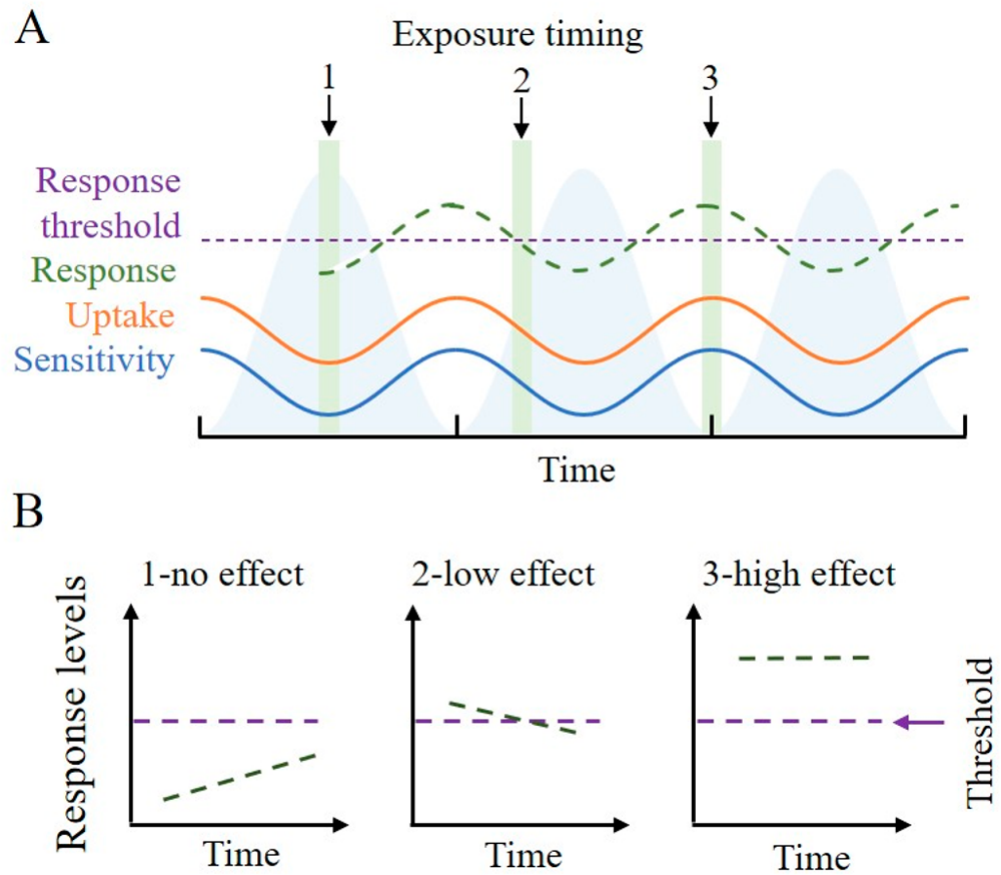
The interplay between physiological rhythms, organismal responses, and the timing of chemical exposure influences their combined impact. (A) Physiological rhythms (shaded blue areas in the background) regulate the uptake kinetics (orange line) and metabolic sensitivity (blue line) in a range of organisms, including plankton and humans. Together, these traits can affect the response of organisms (green dashed line) and, depending on the timing of chemical exposures (green bars), also result in organism-specific toxicity thresholds (purple dashed line) being surpassed. (B) Depending on the interplay between the timing of chemical exposure and physiological rhythms organisms experience a spectrum of effects. No effect (1) occurs when chemical exposure occurs during a time window when the physiologically mediated interaction between uptake and sensitivity has a combined response that falls below organism-specific thresholds. A low effect (2) can occur when exposure to a chemical occurs during a time window when uptake and sensitivity have a combined response that is close to organism-specific thresholds. Finally, a high effect (3) can occur during a time window when the combination of uptake and sensitivity result in a response that surpasses organism-specific thresholds during chemical exposure.

The base of aquatic ecosystems is composed of photosynthetic primary producers (phytoplankton) and their microscopic animal consumers (zooplankton). Phytoplankton sense daily light transitions and adapt their physiology in order to ensure optimal functioning and protection of the photosynthetic apparatus, and zooplankton utilize circadian rhythms to coordinate their diel vertical migration (the largest synchronous movement of biomass on our planet). Both plankton groups thus use physiological rhythms to help align individual metabolic and behavioral processes to environments, a choreography that collectively affects our planet’s ability to capture carbon and sustain food webs. Despite the importance of rhythms for plankton, however, we know little about what happens when plankton become exposed to stressors (e.g., pollution) during specific physiological time windows. A first step is to consider the time scales experienced by plankton. Conceivably, the generation time of fast growing plankton (e.g., certain diatoms under ideal conditions) is less than a day, which implies that typical diel rhythms (e.g., the regulation of photosynthesis) and interrelated changes in physiological “sensitivity” only overlap briefly with a given set of stressors.^1^ Yet, for other slower growing organisms, that is, most plankton under nonideal conditions, generation times span several days and here stress exposures could conceivably overlap with windows of physiologically mediated sensitivity and, critically, surpass organism-specific thresholds (Figure 1A). If true, this implies that the timing between rhythms and stress exposure has significant influence on the outcome of their combined impact (Figure 1B). In its simplest form, the effect of “rhythms” and stress exposure could simply cancel each other out, but more complex situations arise when stressors and rhythms are out-of-phase, potentially resulting in responses that are comparatively higher or lower, or even cause the disruption of physiological rhythms themselves, for example, as shown in freshwater plankton exposed to road salt.^2^ To start addressing this complex interplay in an ecotoxicological context, we must develop high-throughput experimental approaches and accompanying analytical frameworks that can assess interactive impacts on plankton.

## Rhythms That Determine Human Health

Just like plankton, physiological processes in humans and animals are also rhythmic and mediated by external signals. Most prominently, 24 h circadian rhythms are entrained by external zeitgebers such as light and food intake and regulated via the master circadian clock in the suprachiasmatic nucleus of the hypothalamus and peripheral clocks. Molecularly, the circadian clock consists of positive (e.g., CLOCK, BMAL1) and negative (e.g., PER, CRY) clock genes that are expressed in an oscillating pattern and regulate the rhythmic expression of thousands of clock-controlled genes, including those involved in the uptake, metabolism, and excretion of environmental chemicals. This implies that the internal dose at potential target tissue(s) is dependent not only on the timing of exposure in relation to the circadian rhythm but also the sensitivity and uptake kinetics of physiological systems during these times (Figure 1A). Notably, the rhythmicity of these systems should be taken into account when using their products as markers of toxicity, such as changes in the transcriptomic landscape^3^ or epigenetic patterns, which have become of interest as stable markers of adverse effects but have recently been found to exhibit circadian patterns.^4^ Finally, and akin to plankton, chemical exposure in humans can also affect the circadian rhythms themselves, implying that changes in typical effect markers (hormone levels, mRNA expression, DNA methylation) could reflect impacts of a chemical on the circadian rhythm rather than on investigated targets. The diurnal rhythm is one example of a phenomenon that might affect the temporality of chemical exposures. Others are monthly or seasonal, such as the menstrual cycle. In animal experiments, this is sometimes considered by timing exposure and sacrifice according to the diurnal and monthly cycle. However, to systematically disentangle the interplay between rhythms and toxicity in humans and other animals, we must conduct experimental time series exposures and engage in continuous monitoring efforts. While this represents an almost infinite task, emerging single-cell methods can couple investigations of rhythmicity to individual asynchronous effects and thus address their relationship within defined exposure windows.

## Assessing the Complexity of Rhythms

We surmise that understanding stress exposure in the context of physiological rhythms will require analytical insights from other disciplines, such as engineering, meteorology, and economics where timing is not an ancillary feature but a fundamental one. In these disciplines, oscillatory time series data are used, for example, to anticipate weather patterns via autoregression integrated moving average models, deep neural network models, or hybrid methods like random Fourier extreme learning.^5^ While these tools are not exactly designed to delineate the interplay between external stressors and physiological oscillations, they provide inspiration to develop new “systems toxicology” approaches that can do so. Key to this will be the identification of features of particular interest, such as time windows where exposure effects are either maximal or minimal (Figure 1B) and which represent the boundary conditions that envelop a spectrum of possible responses. Finally, we advocate that in this context, fast-growing phytoplankton are compelling model systems for the essential development of rapid experimental and analytical approaches.
